# Exposure-QTc modeling of bedaquiline, pretomanid, and clofazimine in adults with tuberculosis

**DOI:** 10.1128/aac.01399-25

**Published:** 2026-03-09

**Authors:** Mahmoud Tareq Abdelwahab, Elin M. Svensson, Andreas H. Diacon, Almarie Conradie, Morounfolu Olugbosi, Rodney Dawson, Gary Maartens, Paolo Denti

**Affiliations:** 1 Division of Clinical Pharmacology, Department of Medicine, University of Cape Town 71984, Cape Town, South Africa; 2 Department of Pharmacy, Pharmacology and Toxicology, Radboud University Medical Center 6034, Nijmegen, the Netherlands; 3 Department of Pharmacy, Uppsala University 8097, Uppsala, Sweden; 4 Task 728678 , Bellville, South Africa; 5 Global Alliance for TB Drug Development 486654, New York, New York, USA; 6 Division of Pulmonology and Department of Medicine, University of Cape Town Lung Institute 108145, Cape Town, South Africa; Houston Methodist Hospital and Weill Cornell Medical College, Houston, Texas, USA

**Keywords:** tuberculosis, bedaquiline, pretomanid, clofazimine QT interval prolongation, PK/PD modeling

## Abstract

Drug resistance (DR) poses a critical challenge to global efforts to manage tuberculosis (TB). Limited information is available on the combined cardiotoxicity of bedaquiline (BDQ), pretomanid (Pa), and clofazimine (CFZ), key drugs in current DR-TB treatment regimens. All of those prolong the QT interval. We aimed to describe this interaction to predict possible toxicities with established and novel dosing regimens to identify patients at higher risk of QT prolongation. The data for this analysis were obtained from an early-bactericidal activity study in drug-susceptible-TB of several TB drugs. We developed a competitive interaction model to evaluate combined concentration-QTc effect of all three drugs. The model was developed using ECG-matched PK measurements (105 patients, 2,062 observations). The model identified a maximum QT increase by all three drugs of 44.5 ms with respective EC50 values for BDQ, Pa, and CFZ of 0.57, 0.903, and 26.9 mg/L. For patients aged 70 years with non-black ancestry, at risk of increased BDQ exposure, simulations showed that 39.1% had a drug-induced QT change >30 ms after the loading period; this was 29.4% for following BDQ 400 mg daily regimen (part of UNITE4TB program). Reassuringly, no simulations resulted in a QTcF >500 ms, and less than 1% exceeded 480 ms or a change from baseline of >60 ms. We present a joint concentration-QT model of BDQ, Pa, and CFZ. The competitive interaction model serves as a tool to predict possible combined exposure-induced QT prolongation of these drugs in different dosing regimen and identify patients at high risk of significant QT-prolongation.

## INTRODUCTION

Tuberculosis (TB) remains the leading cause of death by infection (1). The occurrence of drug resistance (DR) to TB medications poses critical challenges to its management. In 2022, TB was the largest drug-resistant airborne disease (2). Current regimen recommended by WHO against DR-TB involves treatment regimens of 6 to 9 months, and sometimes up to 18 months if a shorter regimen cannot be given (3). Bedaquiline combined with pretomanid, linezolid, and moxifloxacin (BPaLM) is the recommended treatment regimen against multidrug-resistant/rifampicin-resistant (MDR/RR-TB) (4). The pharmacokinetics of bedaquiline are characterized by slow accumulation and a long terminal half-life (>5 months) (5–7). Bedaquiline’s main metabolite (BDQM2) is associated with QT interval prolongation (8, 9). Pretomanid pharmacokinetics have been described as linear with a half-life of 19 h (10, 11). Pretomanid is also associated with mild QT interval prolongation (12). Clofazimine is a repurposed drug for DR-TB. Clofazimine pharmacokinetics follow slow accumulation with a long half-life (up to 30 days) (13). It also has been linked to a significant QT interval prolongation (14, 15). Clofazimine is being evaluated in novel short dosing regimens against drug-susceptible TB (DS-TB) (16).

Most of the TB drugs with QT-prolonging effect exert their action by interacting mostly with the hERG channel (17). Drugs such as bedaquiline, delamanid, pretomanid, and clofazimine are known to inhibit the hERG potassium channel causing QT-prolongation (18, 19).

Limited information is available on the combined QT-prolongation effects of these drugs when co-administered. In this study, we aimed to identify and describe pharmacological drug-drug interactions to predict the extent of QT prolongation in different novel dosing regimens.

## MATERIALS AND METHODS

Data included in the current analysis were obtained from a 14-day phase 2A study of early bactericidal activity (EBA) of clofazimine, alone or in combination with bedaquiline or pretomanid, and treatment arms with pyrazinamide only (PZA) or standard anti-TB treatment (isoniazid [H], rifampicin [R], Z, ethambutol [E]; HRZE), respectively. Details of the study and drug assays have been published previously (14, 20). In summary, the study enrolled treatment-naïve adults with drug-susceptible pulmonary tuberculosis (DS-TB). The patients were randomized into one of six treatment arms or a control arm. Bedaquiline was administered as 400 mg on day 1, 300 mg on day 2, and 200 mg on days 3–14. Clofazimine was administered as 300 mg for 3 days, followed by 100 mg until day 14. Pretomanid was administered as 200 mg daily for 14 days. Triplicate 12-lead ECGs were collected at pre-specified nominal time points matched to pharmacokinetic (PK) sampling windows on the day before treatment initiation and on Days 1, 2, 3, 8, 14, and 28. Assessments included pre-dose, 5-, and 10-h post-dose. Plasma PK samples were obtained at the same times as the first of the triplicate ECG after the initiation of treatment, and the concentrations of bedaquiline, BDQM2, pretomanid and clofazimine, and pretomanid were available. The sampling schedule was designed to capture the full range of exposures, including the expected peak concentrations of the three drugs, which occur approximately 5 h post-dose. The ECG and PK measurements collected at 5 h post-dose, thus targeting the expected peak concentrations, while pre-dose measurements captured the trough levels.

### Model development

The raw QT data were corrected for heart rate correlation using Fridericia formula (QTcF). The QTcF data were analyzed using a model previously developed on a subset of the current study data. The model characterized the QTcF prolongation effect of clofazimine monotherapy. The model included parameters for baseline QTcF, diurnal variation, and clofazimine drug effect (14). In the current work, we extended the previous model to account for a long-term, non-drug-related change in the QTc interval over time and termed as secular trend (12). The diurnal variation model was developed using pre-treatment data from all study arms and post-treatment data from the arm without QT prolonging drugs (PZA and HRZE treatment arms). Information on diurnal variation available in literature (21) was incorporated as priors to stabilize the estimation of diurnal parameters, using the $PRIOR functionality in NONMEM (22). We used a weakly informative prior with around 30% variance which allows the model to also use the information in the current study data.

Observed drug concentrations were used to investigate exposure-QTcF relationships. The exposure-QTcF model development was built sequentially; we estimated clofazimine drug effect first as the only mono-therapy arm of a QT-prolonging drug, followed by study arms containing combinations with bedaquiline and pretomanid. Different relationship shapes (linear, Emax, and sigmoidal Emax) were explored to describe the relationship between drug concentrations and the QTcF. We also tested for pharmacodynamic drug-drug interactions among the three drugs using different approaches. First, we tried an empirical approach by estimating an interaction parameter *β*, shown in equation 1 below.


(1) $$DE_{ABC}=DE_{A}+DE_{B}+DE_{C}+\beta \cdot DE_{A}\cdot DE_{B}\cdot DE_{C}$$

Where DE_A_, DE_B_, and DE_C_ are the effects of drug A, B, and C, respectively.

**Fig 1:**
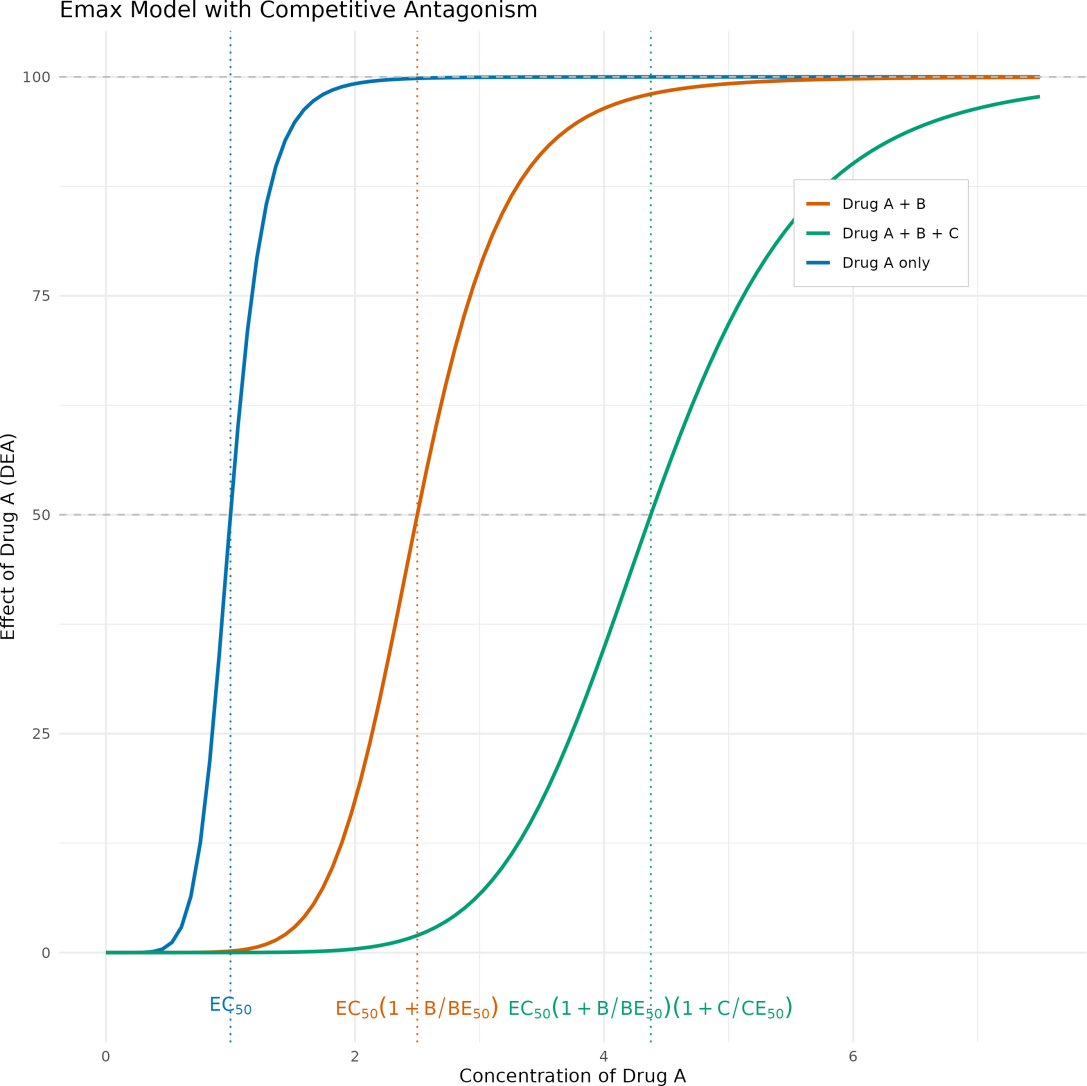
:An example showing competitive antagonism whereby the impact of drug B and drug C shifts the curve of the effect of drug A to the right, thus decreasing its potency. EC50 values of 1, 2, and 4 for drugs A, B, and C and median concentration for drugs B and C of 3 were chosen to generate this figure.


We also tried a more mechanistic approach using a competitive interaction model (equation 2) in which the concentrations of the companion drugs modulate each other’s potency (EC50), as depicted in Fig. 1.

(2) $$\begin{array}{rl} DE_{A} & =\frac{E_{max}\cdot {\text{Conc}}_{A}}{EC_{50,A}\cdot \left(1+\frac{{\text{Conc}}_{B}}{EC_{50,B}}\right)\cdot \left(1+\frac{{\text{Conc}}_{C}}{EC_{50,C}}\right)+{\text{Conc}}_{A}}\\ DE_{B} & =\frac{E_{max}\cdot {\text{Conc}}_{B}}{EC_{50,B}\cdot \left(1+\frac{{\text{Conc}}_{A}}{EC_{50,A}}\right)\cdot \left(1+\frac{{\text{Conc}}_{C}}{EC_{50,C}}\right)+{\text{Conc}}_{B}}\\ DE_{C} & =\frac{E_{max}\cdot {\text{Conc}}_{C}}{EC_{50,C}\cdot \left(1+\frac{{\text{Conc}}_{A}}{EC_{50,A}}\right)\cdot \left(1+\frac{{\text{Conc}}_{B}}{EC_{50,B}}\right)+{\text{Conc}}_{C}} \end{array}$$

The competitive interaction model relies on the assumption that the analytes bind to a common receptor (with different affinity) and have a common mechanism of action to induce QT prolongation, so the maximal effect (Emax) is assumed to be shared (i.e., the same) across all three drugs, but different EC50 values are allowed for each drug. Bedaquiline effect on the QT was assumed to be driven by the BDQM2 concentrations (8), while for the pretomanid and clofazimine, it was driven by the parent molecule.

We tested covariates in a model developed using only pre-treatment and PZA and HRZE arms data then re-validated our findings using the full data set (data from other treatment arms). The following covariates were tested on the baseline QTcF and circadian model parameters: age, sex, body weight, fat-free mass, and electrolyte known to affect QT concentrations (calcium, potassium, and sodium).

### Simulations

We used the parameters from the final model to explore the risk of QTcF prolongation when using bedaquiline, pretomanid, and clofazimine either in combination or as monotherapy. We simulated the following regimens: bedaquiline as 400 mg for 2 weeks followed by 100 mg daily (as used in UNITE4TB BDQ dosing regimen, [23]), bedaquiline as 200 mg for 8 weeks followed by 100 mg daily along with pretomanid dose of 200 mg daily (BPaL [24]), and finally clofazimine as 100 mg daily. Additional clofazimine dosing regimen simulations are provided in the supplementary appendix. All simulations were done up to 26 weeks of dosing. We performed a simulation of 100,000 replicates using final parameter estimates of a typical tuberculosis patient in our cohort of 56 kg with a fat-free mass of 43 kg and baseline albumin level of 3.65 g/dL. We also evaluated the impact of ethnicity and age on regimens containing bedaquiline (5). The simulations considered the between-subject and inter-occasion variabilities in the parameters of the pharmacokinetic models and only between-subject variability in the pharmacodynamic model (5, 10, 13, 25). The simulated QTcF and (change from baseline) ΔQTcF were summarized according to The International Council for Harmonisation of Technical Requirements for Pharmaceuticals for Human Use (ICH) guidelines, which recommend the following different limits and cut-off points when analyzing QT/QTc interval prolongation data: Absolute QTc interval prolongation of >450, >480, and >500 ms or a change in QTc from baseline >30 and >60 ms, respectively (26).

## RESULTS

### Demographics

Summary of the baseline demographics of the patients included in the current analysis is listed in Table 1. One hundred and five patients provided 2,062 ECGs, of which 1,089 had matching PK information (bedaquiline, pretomanid, and clofazimine plasma levels), and the remaining 973 ECG measurements were pre-treatment data and arms without QT-prolonging drugs (PZA and HRZE).

**TABLE 1:** Clinical characteristics of all patients included in the current analysis^*a*^

Characteristic	Median (IQR)/*n* (%)
Age, year	30 (23–40)
Males	65 (61.9)
Living with HIV-1	12 (11.4)
Total body weight, kg	53.8 (46.9–60.4)
FFM, kg	42.7 kg (27.3–64.8)
BMI, kg/m^2^	19.4 (17.8–21.3)
Overall-Baseline QTcF (ms)	390 (379–401)
Predose QTcF (ms)	391 (380–402)
5-h QTcF (ms)	390 (377–398)
10-h QTcF (ms)	388 (378–397)
Overall-Baseline heart rate (bpm)	89.3 (77.4–100)
Predose HR (bpm)	89.3 (77.0–101)
5-h HR (bpm)	89.7 (80.3–99)
10-h HR (bpm)	88.3 (79.7–98)

^*a*^ Fat-free mass (FFM), body mass index (BMI), beat per minute (bpm).

### Model development and final parameters

Three oscillators functions best described the circadian model after relying on prior functionality in NONMEM to stabilize the estimation process. A significant secular trend effect was identified (dOFV −54.3, 2 df, *P*< 0.01). The secular trend was best described by an exponential function reaching a maximum QTcF increase by 6.33 (4.34–8.79) ms with a half-life of 1.04 (0.58–2.29) weeks. The impact of circadian variation on the QTcF baseline and the magnitude of secular trend with time are depicted in supplemental material. Age and potassium levels were identified as significant covariates affecting baseline QTc in the forward selection process, but only age was retained after the backward elimination process (dOFV −14.1, *P*< 0.01).

**Fig 2:**
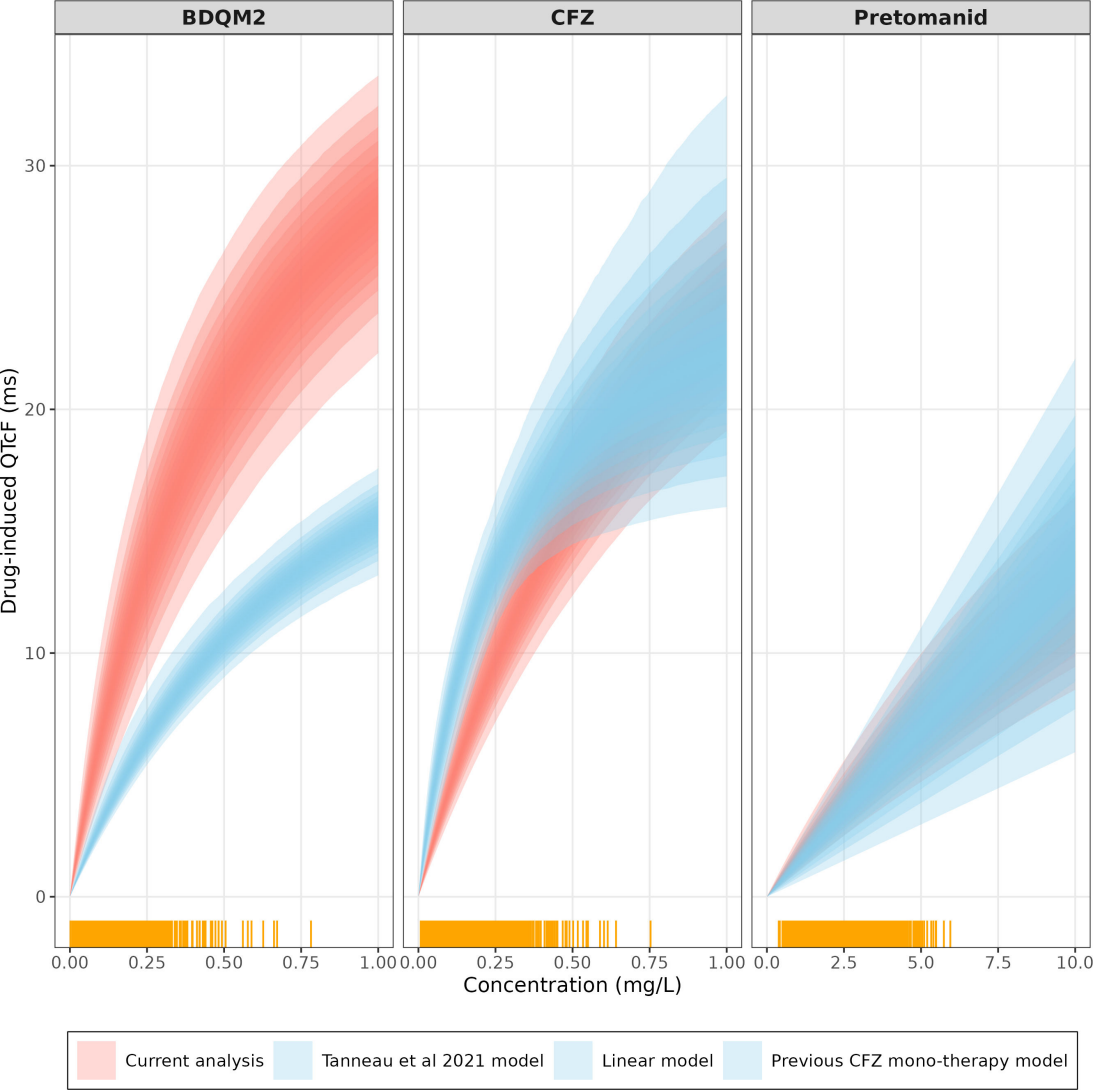
BDQM2, CFZ, and Pretomanid-induced QTcF effects. Current model (red) for each of the drugs (assuming no interaction) vs BDQM2 QTcF effect as predicted by Tanneau et al. (8), CFZ monotherapy (14), and linear pretomanid model identified during model development. Yellow bars on the *x*-axis represent available observations. Shaded areas represent confidence intervals based on the uncertainty of final population parameters.

To describe the effects of BDQM2, pretomanid, and clofazimine jointly, the competitive-inhibition interaction model was found to perform best among tested models in terms of plausible parameter estimates and scientific rationale. The exposure-QTcF effect of BDQM2 and clofazimine was found to follow Emax relationship even when these were estimated separately during model development, so they were easy to integrate into the joint competitive inhibition model. On the other hand, the exposure-QT effect for pretomanid was best described via linear effect (given observed range of concentrations). Pretomanid effect was integrated into the combined model with BDQM2 and clofazimine via Emax relationship and usage of informative prior on the EC50 of pretomanid. Figure 2 shows the linear QT effect of pretomanid within the observed exposure level overlaid by the nonlinear Emax relationship, and additional details are provided in the supplementary.

The maximal drug effect was found to be 44.5 (38.3–53.5) ms with EC50 of 0.57 (0.28–1.26), 0.903 (0.563–1.61), and 26.9 (15.2–51.2) mg/L for BDQM2, clofazimine, and pretomanid, respectively. The dOFV was not significantly different in a model where the QT-drug effects were estimated separately (driven by additional Emax parameter(s)) and the current model. Consequently, the current model was selected based on its fewer estimable parameters and the principle of model parsimony. We also estimated between-subject variability on the EC50(s) for both BDQM2 and clofazimine; however, estimating between-subject variability on the shared Emax parameter did not result in model fit improvement. Final parameter estimates are summarized in Table 2. Visual predictive check showing adequate model fit to the data is depicted in supplementary material.

**TABLE 2:** Final model parameters and confidence intervals

Parameter description	Typical value (95% CI)^*a*^
QTcF base (ms)	393 (390–396)
Diurnal model parameters^*b*^	
24-h cycle amplitude (%)	1.13 (0.714–1.6)
12-h cycle amplitude (%)	0.586 (0.317–0.851)
6-h cycle amplitude (%)	0.289 (0.174–0.457)
24-h cycle acrophase (h)	3.32 (2.63–4.01)
12-h cycle acrophase (h)	3.54 (2.6–4.54)
6-h cycle acrophase (h)	5.14 (3.93–5.84)
Time effect model parameters	
QTc_ss_ (ms)	6.33 (4.34–8.79)
*T*_1/2_ (weeks)	1.04 (0.58–2.29)
Age-effect (ms per year)^*c*^	0.472 (0.27–0.685)
Drug-effect parameters	
*E*_max_ (ms)	44.5 (38.3–53.5)
EC_50_ BDQM_2_ (mg/L)^*f*^	0.57 (0.28–1.26)
EC_50_ CFZ (mg/L)^*f*^	0.903 (0.563–1.61)
EC_50_ Pa (mg/L)^*d*^^,^^*f*^	26.9 (15.2–51.2)
Between-subject variability (%)^*e*^	
QTcF base (%CV)	3.4 (2.99–3.92)
QTc_ss_	81.5 (55.5–108)
CFZ EC50	92 (53.5–133)
BDQM2 EC50	168 (129–223)
Residual variability	31.1 (25.9–37.1)
Residual error model	
Additive error (ms)	8.59 (8.06–9.17)

^*a*^ 95% confidence intervals obtained with Sampling Importance Resampling (SIR).

^*b*^ Priors were used to guide the estimation of the parameters, and priors were obtained from reference 21.

^*c*^ Centered at age of 37.5.

^*d*^ Prior of the EC50 value was set to 25.3 mg/L and assuming uncertainty on the prior of ~30%. The value was obtained by constructing a QT linear model based on observed concentrations in the study and previous reports (1.57 ms/(mg/L)) and then solving for the EC50 value that was provided similar prediction to the linear model within the observed pretomanid concentrations assuming shared Emax of 44.2 (ms).

^*e*^ Between-subject variability (BSV) was assumed to be log-normally distributed and reported as approximate %CV.

^*f*^ EC_50_ was calculated as a fraction of *E*_max _as the two parameters are positively correlated and to stabilize the model estimates.

### Simulations

**Fig 3:**
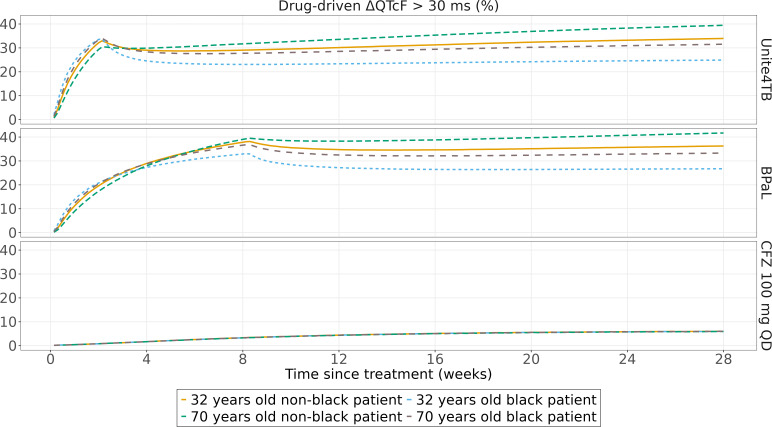
Proportions of simulated replicates with ΔQTcF above 30 ms with different dosing regimens for a 56 kg weight with a fat-free mass of 43 kg and baseline albumin level of 3.65 g/dL TB patient.

Monte-Carlo simulations for the suggested dosing regimen are shown in Fig. 3. The simulations were performed for an *in-silico* male patient either 32- or 70-year-old, either black or non-black. The simulations show the percentage of simulation replicates exceeding a pre-defined threshold; for QTcF, it was defined as 480 ms and 30 ms for ΔQTcF (26). The percentage of replicates for which the drug-driven change from baseline was above 30 ms (ΔQTcF >30) was 29.4% and 39.1% at the end of the loading dose period (2 weeks for UNITE4TB bedaquiline dosing regimen and 8 weeks for BPaL dosing regimen), respectively. Non-black 70 years old patient was chosen to reflect patients at higher risk of experiencing high BDQM2 exposure driven by lower clearance and therefore significant QTcF prolongation (27). After 28 weeks of dosing, these numbers increased to 39.7% and 41.7%. None of the simulation replicates exceeded 60 ms ΔQTcF or only very small fraction for 480 ms QTcF. The proportions for other scenarios are shown in Table 3.

**TABLE 3:** Percentage of simulations replicates exceeding ∆QTCF > 30 (ms) per race (70 years of age) group for different dosing regimen

Dosereg	Time^*a*^ (weeks)	Race	Drug-driven ΔQTcF > 30 ms (%)
BPaL	8	Non-Black	39.1
	8	Black	36.3
	28	Non-Black	41.7
	28	Black	33.3
Unite4TB BDQ	2	Non-Black	29.3
	2	Black	33.4
	28	Non-Black	39.5
	28	Black	31.5

^*a*^ Data at end of loading dose period (8 weeks for BPaL and 2 weeks for Unite4TB BDQ dosing regimen).

## DISCUSSION

In this work, we report on the joint exposure-QT relationship of bedaquiline, pretomanid, and clofazimine. The combined effects were best described as competitive antagonist and not additive. The data were obtained from a 14-day EBA study in DS-TB patients. Additionally, time on treatment or secular trend was also identified in this cohort where the QT increased non-linearly reaching a plateau after 6 weeks of treatment. None of the simulation’s replicates exceeded absolute QTcF of 500 ms, and a very small proportion exceeded 480 ms threshold. Lastly, proportions of simulation replicates of patients with non-Black ancestry that exceeded 30 ms increase in ∆QTCF threshold are mainly driven by increased accumulation of BDQM2 levels.

These results shed additional insights into the combined pharmacological interaction when these drugs are co-administered, and separate exposure-QT relationships of these drugs have been previously characterized (8, 12, 14). Research suggests that the combination of drugs with QTc-prolonging properties does not invariably lead to an additive increase in the QTc interval (28). This observation points toward the potential involvement of non-additive interaction mechanisms, such as competitive antagonism, particularly when drugs share the same target (28, 29).

Our analysis could describe the combined QT effect of these drugs using a competitive antagonist approach. In this approach, competitive antagonists change the location of the inflection point of the Emax curve to the right, thus decreasing the “potency” of the other drugs to increase QT so that a higher drug concentration is required to achieve the same effect in the presence of competitive antagonist (30). When testing alternative models during the analysis, having a separate exposure-QT model for all three drugs did not perform significantly better in terms of fit to the data and the parameter estimates obtained were implausible and incompatible with previous findings about the QT prolonging effect of these drugs.

Previous analyses reported BDQM2 EC50 values ranging from 0.451 to 0.855 mg/L with a corresponding Emax ranging from 23.8 to 28.6 ms (8, 9, 31, 32). Our analysis estimated an EC50 for BDQM2 of 0.57 mg/L, in line with the previous values, and a corresponding Emax of 44.4 ms, which is higher than previously reported (Fig. 2). The estimated Emax value is not only driven by BDQM2 concentrations but also determined by pretomanid and clofazimine in the current interaction model. Additionally, the observed difference in Emax could be due to a true difference in the BDQM2 effect, potentially arising from variations in study populations and background medication. However, it is also possible that this difference is a result of bias in how the BDQM2 effect was described in previous work compared to our current analysis due to the absence of a bedaquiline monotherapy arm in our study.

In the case of pretomanid, during model development, we initially used a linear model for its concentration-QT relationship, and we obtained a value of 1.42 ms/(mg/L), which is in a close agreement with the report by Li et al. (12). However, within the range of pretomanid concentrations observed clinically, the nonlinear relationship in our model is consistent with the linear model initially estimated, as shown in supplemental material.

For clofazimine, the current analysis estimated an EC50 of 0.903 mg/L and a (shared) Emax of 44.5 ms. In our previous analysis on the CFZ monotherapy only (14), we estimated an EC50 of 0.281 mg/L and Emax of 28 ms. A possible reason for such difference could be the absence of secular trend effect from the monotherapy model previously published. Figure 2 compares the two CFZ-QT effect models including uncertainty. The estimates from the previous model largely overlap with the current analysis, which has a narrower 95% CI, in line with the larger sample size. Furthermore, the high Emax observed in the current analysis is also driven by the effect of other QT-prolonging drugs included in the interaction model.

One interaction model similar to ours has been applied before. Tanneau et al. described the joint QT effect of BDQ and its main metabolite BDQM2, but the data did not robustly support estimation of the joint effect, which was not significantly better than estimating the QT effect of BDQM2 only (8). In a subsequent paper, the same authors used the competitive interaction model for the QT effect of BDQM2 along with delamanid (31) and estimated a shared Emax for the two drugs. In our analysis, we extended the same model to a third perpetrator drug but also could not estimate a separate Emax for each of the drugs due to the observed concentrations being lower than the range where all three drugs have the same maximal effect.

Absolute QT was used as the dependent variable to preserve the information content of the data when characterizing baseline variability (33). Additionally, it allowed us to investigate and estimate covariate effects on baseline, such as sex and electrolytes, and to estimate any correlation between baseline and secular trend parameters.

Lastly, we estimated a time-dependent increase in the QT interval, reaching a maximum of 6.33 (ms) after around 6 weeks (Supplemental material). This increase in the QTc has been previously reported in literature (9, 12, 31). Patients with active TB often exhibit elevated heart rates at the initiation of treatment. As the treatment becomes effective and the infection is brought under control, these heart rates tend to normalize (12). The magnitude of the secular trend could be related to the correction method applied; prior to the final modeling, we conducted a comprehensive evaluation of multiple correction methods, including Bazett (QTcB), Fridericia (QTcF), Individual Correction (QTcI), and a spline-based correction method. The estimated exponent from our data (approximately 0.41) fell between the fixed coefficients of Fridericia (0.33) and Bazett (0.50). Additionally, spline and individual methods provided only marginal improvements in flattening the QTc-HR slope (34). However, the inclusion of secular trend resulted in improved model-fit in all cases, suggesting other factors contributing to the secular trend. Furthermore, the development of a recently published time-varying correction factors like QTcTBT directly addresses the evolving relationship between QT interval and heart rate during TB treatment (35). The authors suggest that the normalization of heart rate is a key factor influencing the secular trend; however, further research is warranted to refine QT correction methods in TB patients.

We used our model to predict the extent of QT prolongation with other dosing regimens. BDQ exposure is affected by age and ethnicity (5). We explored the impact of these factors on QTcF prolongation following BDQ administration in BPaL and UNITE4TB BDQ dosing regimen.

Of note, BDQ and Pa are administered along with moxifloxacin in the novel BPaLM dosing regimen. Moxifloxacin is known to cause QTcF prolongation effects (36). Thus, the prevalence of patients exceeding the pre-defined QT criteria would eventually be higher and frequent ECG monitoring is warranted. However, it remains unclear whether the competitive interaction observed in our model extends to combinations including moxifloxacin. Therefore, future investigations should prioritize characterizing the effect of each of these drugs over a longer period, especially for bedaquiline and clofazimine and how these drugs prolong the QT interval when administered with other known QT-prolonging drugs, such as moxifloxacin

### Limitations

There are several limitations in the current analysis. The observation period in the current study was up to 28 days, which did not allow us to identify the possible development of any tolerance/desensitization upon chronic drug treatment. We also did not have data on BDQ monotherapy to adequately assess the QT effect of BDQM2, but our estimate of BDQM2 EC50 was not significantly different from previous reports.

The current data alone did not support robust characterization of circadian rhythm, but we incorporated available literature information as weakly informative priors to stabilize the baseline oscillation component of the model. Crucially, these priors were intended strictly to facilitate numerical stability and prevent convergence failure. Consequently, the final posterior parameter estimates—particularly those characterizing the drug-concentration effects—were driven primarily by the observed study data rather than the prior assumptions

While the inclusion of BSV on Emax is desirable for risk assessment, this parameter was not identifiable in our data set (estimates collapsed to zero). Consequently, our simulations may rely on variability in potency (EC50) and exposure to capture the range of patient risk, potentially underestimating extreme outliers in intrinsic channel sensitivity.

Finally, the secular trend estimated in our analysis is associated with large uncertainty. The literature reports different estimates, for example, Minocha et al. reported time-drift that resulted in reduction in QT up to 5 ms with a half-life of 1.4 days (21). Others reported an increase of up to 7 ms with longer half-life (8) although Minocha et al. study was conducted in healthy volunteers, while Tanneau et al. was performed in MDR-TB patients.

### Conclusion

In conclusion, this analysis suggests that BDQ, Pa, and CFZ may compete for the same target, resulting in a non-additive increase in the QTc interval. Specifically, the model indicates that in the presence of other competitive antagonists, a higher concentration of each drug is required to achieve the same level of QT prolongation. We also highlighted that patients with increased BDQ exposure, especially older patients with non-Black ancestry, are at higher risk of exceeding the pre-defined threshold of ∆QTCF 30 ms as defined by ICH. Consequently, careful ECG monitoring for these patients during the initial phase of treatment and during the maintenance period for patients subjected to high BDQM2 accumulation is necessary. Lastly, none of the simulations exceeded ∆QTCF 60 ms or absolute QTcF value of 500 ms.

## SUPPLEMENTAL MATERIAL

Supplemental material aac.01399-25-s0001.docx Table S1; Fig. S1 to S4.

ASM does not own the copyrights to Supplemental Material that may be linked to, or accessed through, an article. The authors have granted ASM a non-exclusive, world-wide license to publish the Supplemental Material files. Please contact the corresponding author directly for reuse.
