# Supplementary material for: Exposure-QTc modeling of bedaquiline, pretomanid, and clofazimine in adults with tuberculosis
Source: Antimicrob Agents Chemother. 2026 Mar 9;70(4):e01399-25. doi: 10.1128/aac.01399-25 (PMC13041299; doi:10.1128/aac.01399-25)
Supplement: Supplemental material — Table S1; Fig. S1 to S4. [file aac.01399-25-s0001.docx]

**Title: Exposure-QTc modelling of bedaquiline, pretomanid and clofazimine in adults with tuberculosis.**

Authors: Mahmoud Tareq Abdelwahab^1^, Elin M. Svensson^2,3^, Andreas Diacon^4^, Almari Conradie^5^, Morounfolu Olugbosi^5^, Rodney Dawson^6^, Gary Maartens^1,7^, Paolo Denti^1^

**Institution:**

1. Division of Clinical Pharmacology, Department of Medicine, University of Cape Town, Cape Town, South Africa.
2. Department of Pharmacy, Pharmacology and Toxicology, Radboud University Medical Center, Nijmegen, Netherlands
3. Department of Pharmacy, Uppsala University, Uppsala, Sweden.
4. Task Applied Science, Bellville, South Africa.
5. Global Alliance for TB Drug Development, New York, USA.
6. Division of Pulmonology and Department of Medicine, University of Cape Town Lung Institute, Mowbray, Cape Town, South Africa
7. Wellcome Centre for Infectious Diseases Research in Africa, Institute of Infectious Disease and Molecular Medicine, Department of Medicine, University of Cape Town, Cape Town, South Africa

## Model development

The model development and covariates inclusion were based on physiological plausibility, inspection of diagnostic plots including visual predictive checks, (1) and significant decreases in the NONMEM objective function value (OFV). Between-subject variability was considered and tested on all parameters assuming log-normal distributions. The statistically significant cut-off for an additional degree of freedom (inclusion of one parameter) was a drop in OFV of at least 3.84 points, corresponding to a p-value of <0.05. We used NONMEM 7.5.1 (ICON PLC, Ireland) and the algorithm FOCE-I for parameters estimation. Pirana, Perl-speaks-NONMEM (PsN) version 5.2.0, and Xpose4 were used to aid the model development process (3, 4). Precisions of the final model parameters were obtained using sampling importance resampling (SIR) available in PsN (5).

Additional clofazimine dosing regimen simulations are provided in the supplementary

### Pretomanid effect as Emax

The estimation of the EC50 of pretomanid was supported by an informative prior. To determine a suitable value for this prior, we transformed the exposure-QT relationship for pretomanid into an Emax function, used the Emax value estimated with BDQM2 and CFZ, and selected a value of EC50 such that the new Emax relationship would overlap with the linear one over the observed range of concentrations.

*Table 1 Percentage of simulations replicates exceeding ∆QTCF > 30 (ms) and QTCF > 450 (ms) per race and age group for different dosing regimen.*

| Dose Regimen | Time (weeks) | Race | Age (years) | dQTcF >30ms (%) | QTcF >480ms (%) |
| --- | --- | --- | --- | --- | --- |
| BPaL | 8 | 1 | 32 | 37.81 | 0.27 |
|  | 8 | 1 | 70 | 39.03 | 0.27 |
|  | 8 | 2 | 32 | 32.91 | 0.21 |
|  | 8 | 2 | 70 | 36.6 | 0.25 |
|  | 28 | 1 | 32 | 36.24 | 0.26 |
|  | 28 | 1 | 70 | 41.68 | 0.29 |
|  | 28 | 2 | 32 | 26.74 | 0.17 |
|  | 28 | 2 | 70 | 33.3 | 0.23 |
| CFZ 100 mg QD | 28 | 1 | 32 | 5.99 | 0.03 |
|  | 28 | 1 | 70 | 5.92 | 0.02 |
|  | 28 | 2 | 32 | 5.98 | 0.02 |
|  | 28 | 2 | 70 | 5.95 | 0.03 |
| Unite4TB BDQ | 2 | 1 | 32 | 32.13 | 0.16 |
|  | 2 | 1 | 70 | 29.34 | 0.13 |
|  | 2 | 2 | 32 | 33.55 | 0.14 |
|  | 2 | 2 | 70 | 33.43 | 0.16 |
|  | 28 | 1 | 32 | 33.95 | 0.24 |
|  | 28 | 1 | 70 | 39.46 | 0.28 |
|  | 28 | 2 | 32 | 24.91 | 0.14 |
|  | 28 | 2 | 70 | 31.53 | 0.22 |
| Race : 1 is non-black ancestry, 2 is black ancestry  Loading period for BPaL is 8 weeks, for Unite4TB BDQ dosing is 2 weeks.  CFZ was dosign 100 mg daily | | | | | |


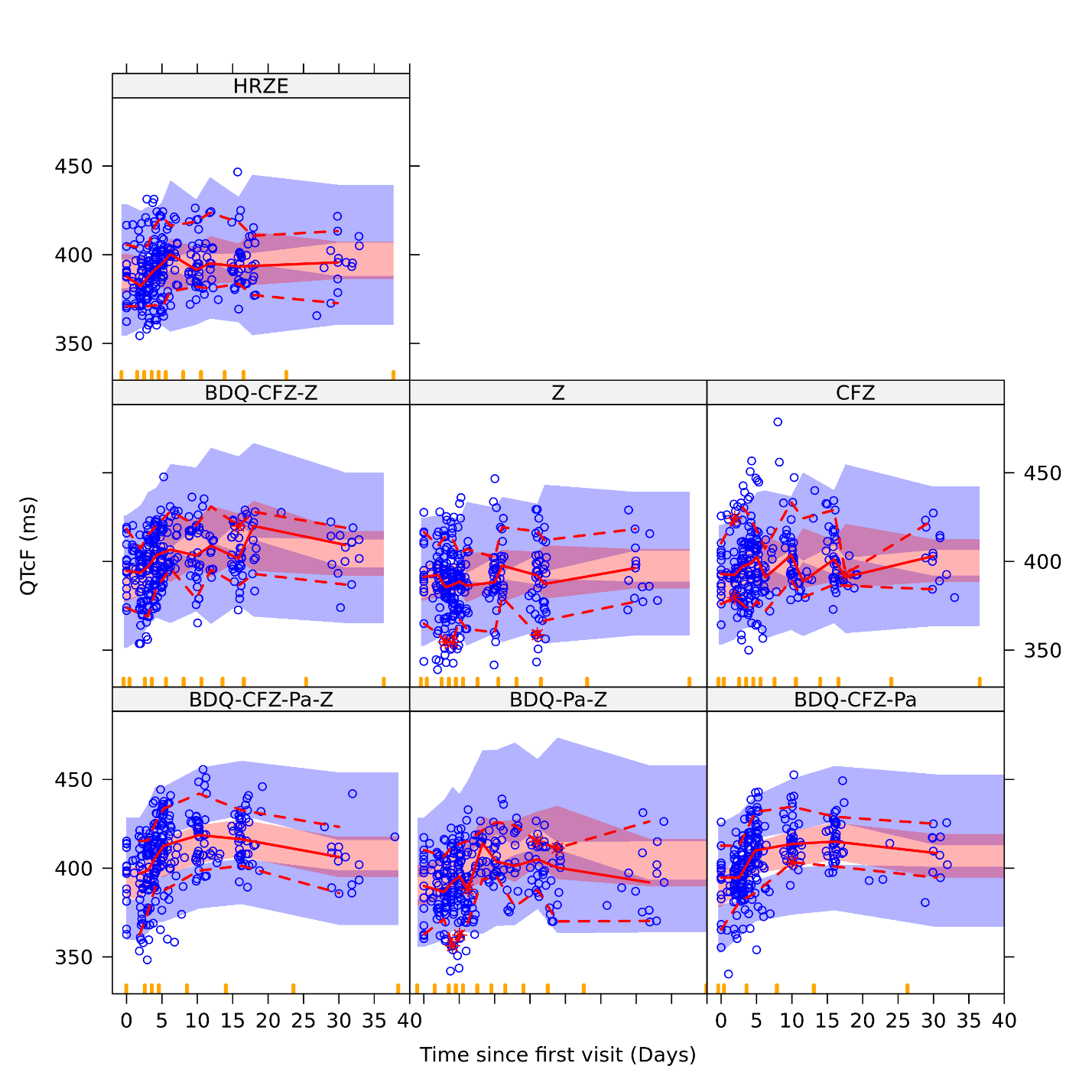


Figure 1: Visual predictive check (VPC) for QTcF (ms) versus time (days) stratified by treatment arm. Circles represent original data; dashed lines are the 10th and 90th percentiles of the original data; solid lines are the 50th percentiles of the original data; while the shaded areas are the corresponding 95% confidence intervals for the same percentiles, as predicted by the model. Vertical yellow ticks on the x axis represent bins for the sampling time points. An appropriate model is expected to have most observed percentiles within the simulated confidence intervals. BDQ=Bedaquiline, Pa= Pretomanid, CFZ = Clofazimine, HRZE = isoniazid [H], rifampicin [R], Z, ethambutol [E] and Z = pyrazinamide.


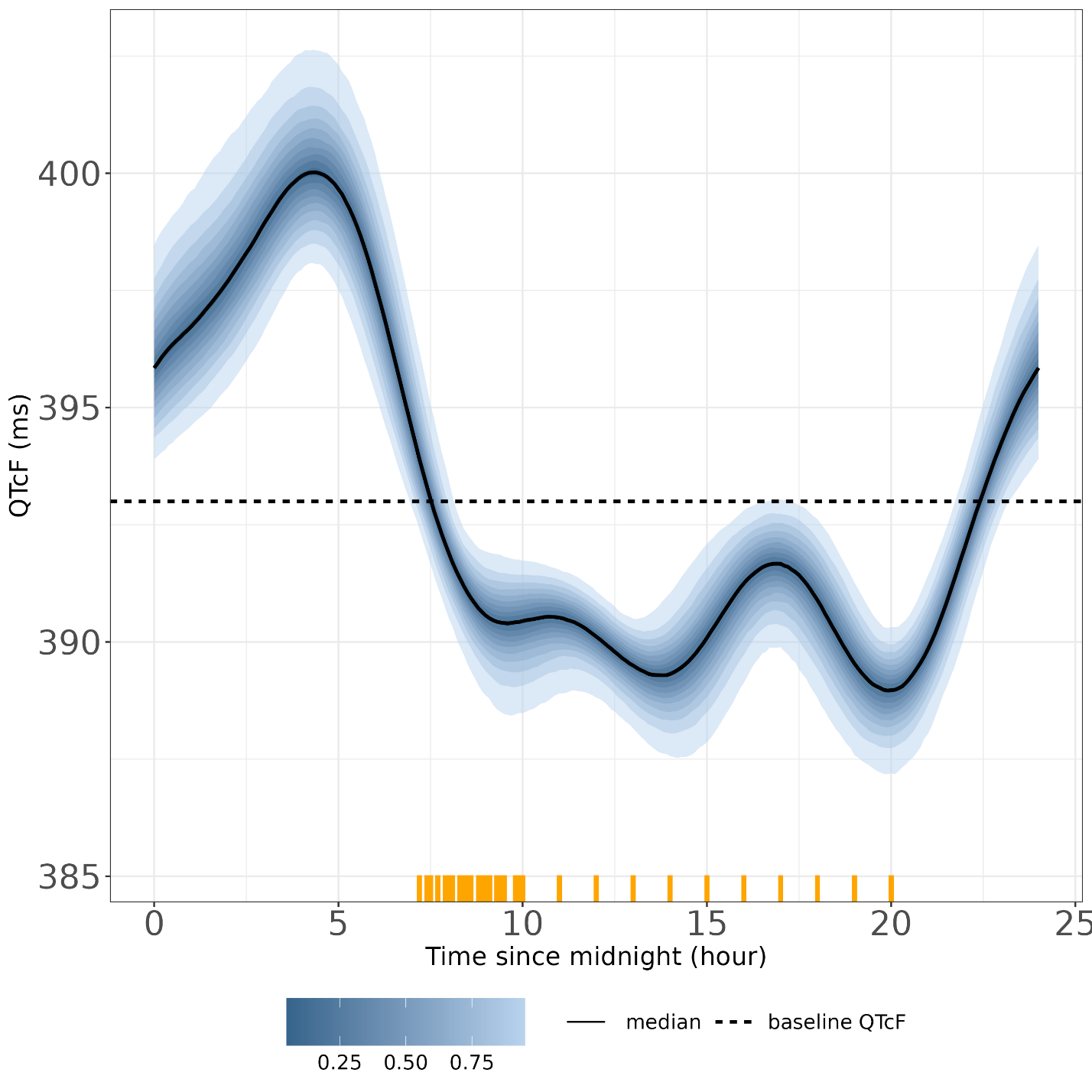


Figure 2 Baseline model accounting for circadian variations in QT vs time since midnight. The solid black line represents the final median parameter estimate, the shaded colored areas reflect the overall uncertainty of the circadian model parameters estimates. Yellow bars on x-axis represent time points where observations are available.


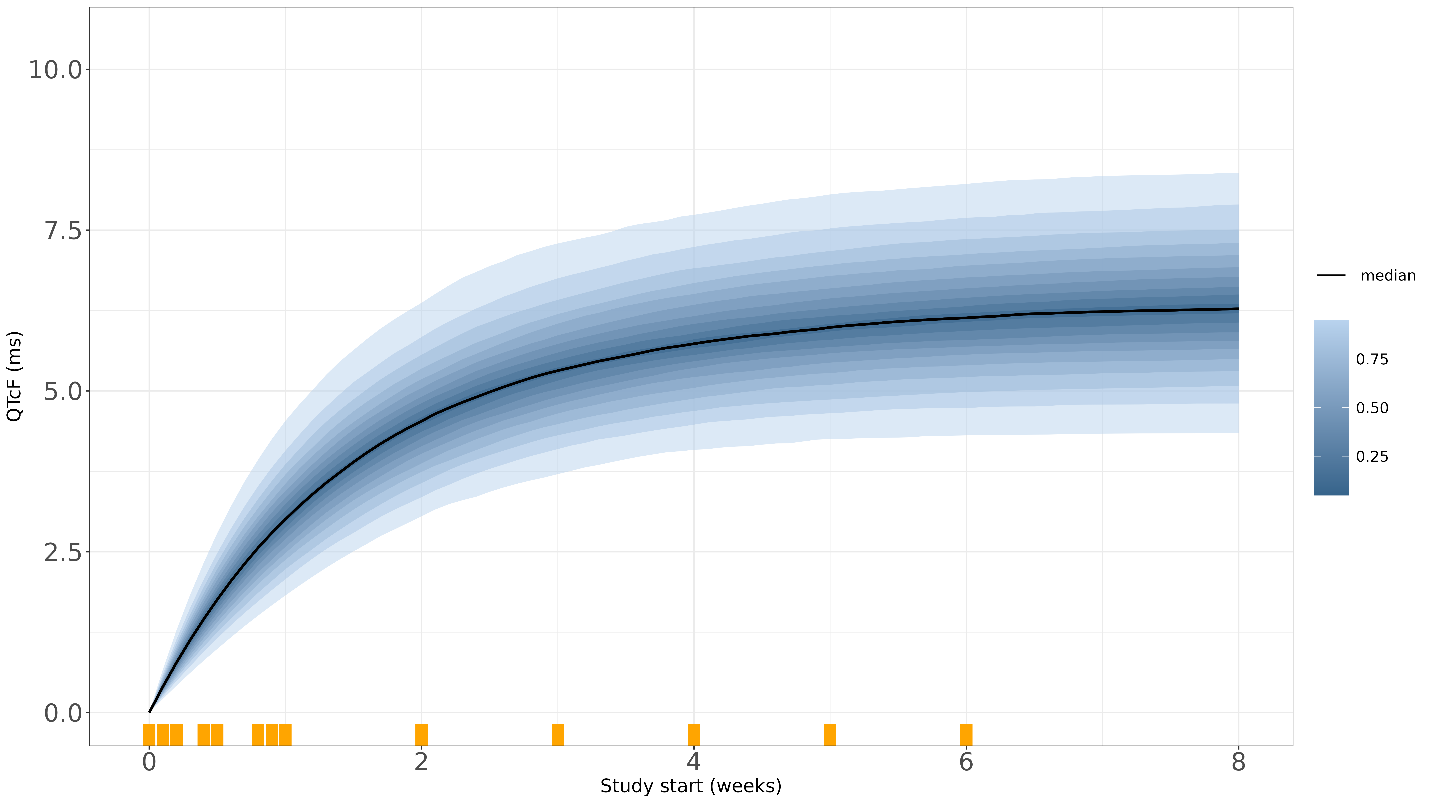


Figure 3: Baseline model showing secular trend over study duration. The solid black line represents the final median parameter estimate, while the shaded colored areas reflect the overall uncertainty or different confidence intervals. Yellow ticks on x-axis denote time points where observations are available.


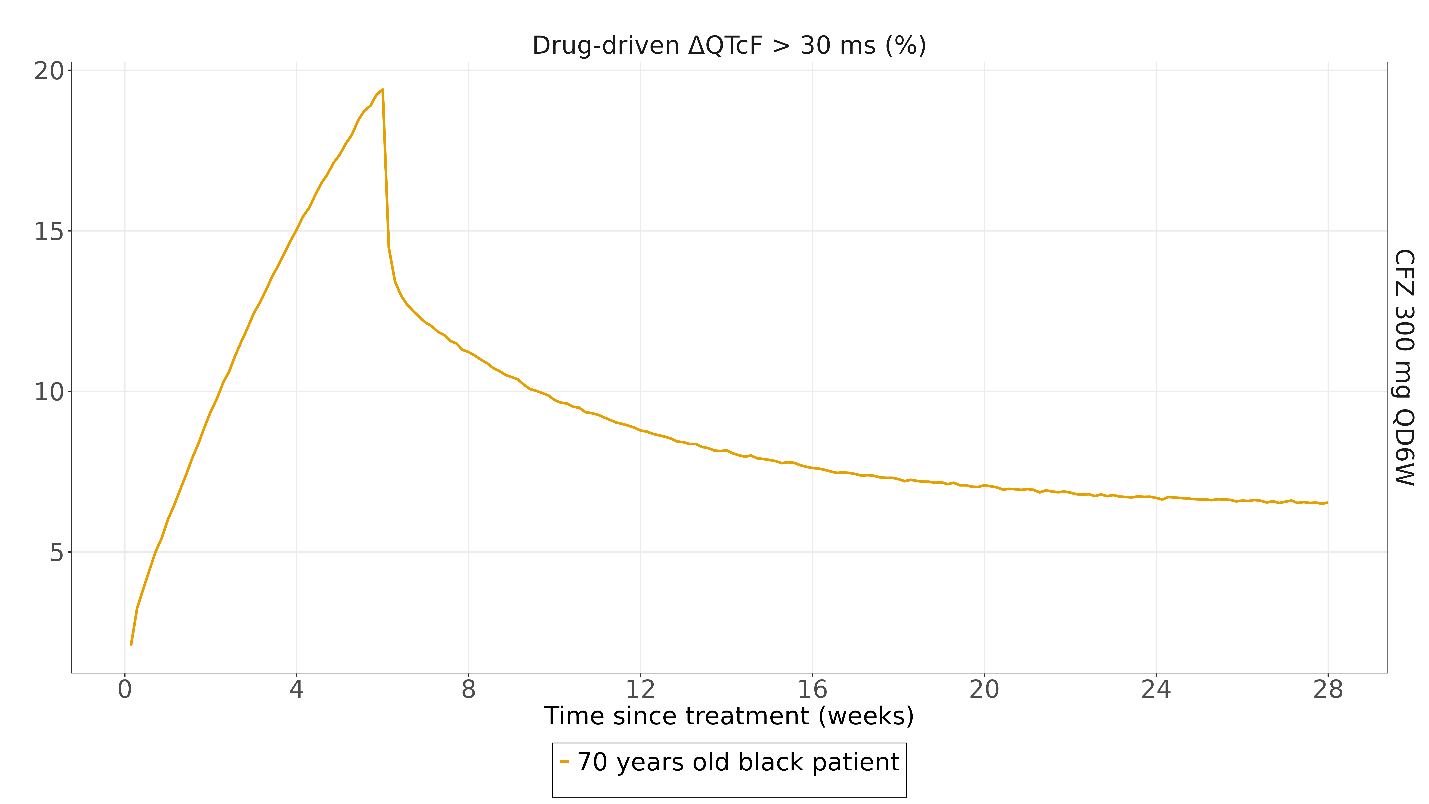


Figure 4: Proportions of simulated replicates with drug-driven ΔQTcF above 30 ms for a 56 kg weight with a fat-free mass of 43 kg TB patient receiving 300 mg of clofazimine loading dose for 6 weeks followed by 100 mg daily.

1. Holford NHG. 2005. The Visual Predictive Check Superiority to Standard Diagnostic (Rorschach) Plots. PAGE Abstr Annu Meet Popul Approach Gr Eur ISSN 1871-6032 PAGE 14 (2005) Abstr738.

2. Beal S, Sheiner L, Boeckmann A, Bauer R (eds). 2020. NONMEM 7.5 Users Guides. (1989-2020). ICON plc, Gaithersburg, MD.

3. Keizer RJ, Karlsson MO, Hooker A. 2013. Modeling and simulation workbench for NONMEM: Tutorial on Pirana, PsN, and Xpose. CPT Pharmacometrics Syst Pharmacol 2.

4. Lindbom L, Ribbing J, Jonsson EN. 2004. Perl-speaks-NONMEM (PsN)—a Perl module for NONMEM related programming. Comput Methods Programs Biomed 75:85–94.

5. Dosne A-G, Bergstrand M, Karlsson MO. 2017. An automated sampling importance resampling procedure for estimating parameter uncertainty. J Pharmacokinet Pharmacodyn 44:509–520.
